# Voluntary EMG-to-force estimation with a multi-scale physiological muscle model

**DOI:** 10.1186/1475-925X-12-86

**Published:** 2013-09-04

**Authors:** Mitsuhiro Hayashibe, David Guiraud

**Affiliations:** 1 INRIA DEMAR Project and LIRMM, UMR5506 CNRS University of Montpellier, 161 Rue Ada, 34095 Montpellier, France

**Keywords:** Muscle model, EMG, Muscle force estimation, Hill model, Multi-scale physiology

## Abstract

**Background:**

EMG-to-force estimation based on muscle models, for voluntary contraction has many applications in human motion analysis. The so-called Hill model is recognized as a standard model for this practical use. However, it is a phenomenological model whereby muscle activation, force-length and force-velocity properties are considered independently. Perreault reported Hill modeling errors were large for different firing frequencies, level of activation and speed of contraction. It may be due to the lack of coupling between activation and force-velocity properties. In this paper, we discuss EMG-force estimation with a multi-scale physiology based model, which has a link to underlying crossbridge dynamics. Differently from the Hill model, the proposed method provides dual dynamics of recruitment and calcium activation.

**Methods:**

The ankle torque was measured for the plantar flexion along with EMG measurements of the medial gastrocnemius (GAS) and soleus (SOL). In addition to Hill representation of the passive elements, three models of the contractile parts have been compared. Using common EMG signals during isometric contraction in four able-bodied subjects, torque was estimated by the linear Hill model, the nonlinear Hill model and the multi-scale physiological model that refers to Huxley theory. The comparison was made in normalized scale versus the case in maximum voluntary contraction.

**Results:**

The estimation results obtained with the multi-scale model showed the best performances both in fast-short and slow-long term contraction in randomized tests for all the four subjects. The RMS errors were improved with the nonlinear Hill model compared to linear Hill, however it showed limitations to account for the different speed of contractions. Average error was 16.9*%* with the linear Hill model, 9.3*%* with the modified Hill model. In contrast, the error in the multi-scale model was 6.1*%* while maintaining a uniform estimation performance in both fast and slow contractions schemes.

**Conclusions:**

We introduced a novel approach that allows EMG-force estimation based on a multi-scale physiology model integrating Hill approach for the passive elements and microscopic cross-bridge representations for the contractile element. The experimental evaluation highlights estimation improvements especially a larger range of contraction conditions with integration of the neural activation frequency property and force-velocity relationship through cross-bridge dynamics consideration.

## Introduction

Any human movement is produced by muscular and skeletal systems controlled by the nervous system. Mechanism of the human body dynamics has been revealed by many biomechanical researches. The general neuromusculoskeletal model of the whole body and its dynamics computation package have been developed and reported [1]. However, the detailed neuromuscular activation system should still be closely analyzed and modeled from microscopic to macroscopic scales. Macroscopic modeling, such as the Hill-type muscle model, is a phenomenological model. Conversely, microscopic modeling is based on muscle physiology, more specifically the dynamics of actin-myosin, allowing it to display a richer response.

Neuromuscular modeling is important for neuroscience to understand how limb movements are smoothly and effectively controlled [2,3]. It is also valuable for clinical applications to be used for quantitative analysis on abnormal muscle activation patterns such as spasticity induced by stroke or cerebral palsy [4]. Moreover, neuroprosthetic control such as functional electrical stimulation (FES) for paralyzed muscles [5,6] can be improved through neuromuscular modeling to optimize the muscle activation control.

Surface electromyography (EMG) is widely used to assess the muscle status in a wide range of clinical fields. The signal generated during a contraction is the summation of the signals of all different recruited motor units (MU) at a given time. Each MU receives information from the central nervous system in the form of action potentials, which are transmitted from the alpha motor neuron to the muscle fibers via the neuromuscular junction. During voluntary contraction, the EMG signal can be seen as the interference pattern of all active MUs, with each MU firing at its own mean frequency with its own timing [7]. The contributions of individual MU cannot be easily recovered from the EMG signal. However, EMG signals still contain precious information about muscle activation and could thus represents the entry of a muscle model. Indeed, the important advantage of EMG usage is that it can account for a subject’s individual activation pattern to estimate muscle force. Moreover, as the EMG signals can be considered for each individual groups of antagonist muscles with their own muscle models, co-contraction can be captured and deeply investigated through the computation of individual force and stiffness. On the contrary, numerical optimization techniques typically do not account for muscular co-contraction.

EMG-based models were already used in many studies to estimate torques around joints [8] or in musculoskeletal models [9,10]. Most of the muscle models used in such studies are based on phenomenological models derived from Hill’s study [11] and summarized by Zajac [12]. The Hill-type model is attractive in biomechanical simulations because of the computational simplicity and easy to calibrate using experimentally measurable variables. It tends to produce accurate estimation results, however in limited ranges of function.

However, recent work [13] performed the validation of Hill model under functionally wider relevant conditions. The authors concluded that the modeling errors were substantial for different firing frequencies and greatest at low motor unit firing rates that are most relevant in normal movement conditions both in voluntary and FES muscle contractions. They pointed out that this could be due to the Hill model assumption whereby muscle activation, force-length and force-velocity properties are considered independently. In their discussion, it was suggested that more physiological coupling between activation and force-velocity properties can be demonstrated in crossbridge models incorporating dependence between physiologically based activation and cross-bridge attachment [13]. The important finding is that Hill modeling errors were large for different firing frequencies and greatest at low motor unit firing rates. In [13], classical linear Hill model was used. Especially for solving the problem of the mismatch at low muscle activation level, a nonlinear conversion was proposed [14] and used by other researchers [9]. This can solve partially the problem, however, there is a still the problem to correspond to the estimations in different contraction speeds. Besides, in Hill-type modeling, the cut off frequency should be carefully chosen depending on the type of task because the envelope of the estimated force is almost defined by the low-pass filtering of the activation. Thus, we aim at proposing multiscale muscle model, which can have more physiological coupling between activation and force-velocity properties by incorporating microscopic cross-bride and dual dynamics pathway for the recruitment / activation level and the calcium triggering the contraction cycle. However, the whole passive structure is based on Hill model as only the contractile element differs.

The first microscopic muscle model was proposed by Huxley [15]. He detailed the interaction of the cross-bridges in a sarcomere in order to explain the force generation. The distinctions between microscopic and macroscopic levels are not absolute; thus a sarcomere model can be used to represent a whole muscle, which is assumed to be a homogeneous assembly of identical sarcomeres. Conversely, the Hill model, which was originally developed for whole muscles, has been used to represent the dynamics of individual sarcomeres within a fiber [16]. The distribution-moment approach of Zahalak [17] is a model for sarcomeres or whole muscle, which is extracted via a formal mathematical Gaussian approximation from Huxley cross-bridge model. This model fills a gap between microscopic and macroscopic levels. Based on Huxley and Hill-type models, Bestel-Sorine [18] proposed an explanation of how the beating of cardiac muscle may take place, through chemical control input connected to the calcium dynamics of cells in muscles. This chemical input triggers the contractile element model.

Complete Huxley type physiological model have a greater repertoire of response, but are computationally complex. Bestel-Sorine representation provides an efficient trade-off between complexity and power of the model expression. It consists in an explicit computation of the first two moments reprenting stiffness and force of the contractile element, triggered by the chemical input as regard the contraction start and stop. Starting with this concept, we first adapted it to the striated muscle model to represent muscle responses under FES [19]. The multi-scale muscle model was applied for voluntary contraction in a preliminary work [20]. In this study, we aim at developing EMG-based muscle model to study voluntary muscle contraction with this multiscale modeling approach compared to Hill approaches, both linear and nonlinear. Indeed, in Hill approach, the dependency on cut-off frequency choice prevents from good performances in a wide range of contraction types. The multiscale approach should improve the performances in particular regarding fast and slow contraction patterns. In addition, we consider that it is of interest to apply the same type of EMG-to-force estimation with a physiologically detailed model and not only with a phenomenological Hill model because the internal biophysical dynamics could shed new light on neuromuscular activation. Using common data sets of isometric muscle contractions, the force estimation results are compared between Hill model approaches and newly proposed approach.

## Methods

### Hill-type muscle model

The transformation from EMG to muscle activation is a dominant process because the estimated muscle force is assumed to be proportional to the muscle activation.

#### EMG processing

The EMG processing method employed in this paper is summarized. For the details, readers can refer to [8,9]. 

1. high-pass filtering of raw EMG using zero-lag 4th order Butterworth filter (30 Hz) to remove motion artifacts

2. wave rectification

3. low-pass filtering with a 2 Hz cut-off frequency using zero-lag 2nd order Butterworth filter

4. normalization with the peak of maximum voluntary contraction (MVC)

There is an alternative method instead of the low-pass filtering of the third step. It is referred to as the mean absolute value (MAV) method. A moving average filter is applied within a fixed time window, which is equivalent to low-pass filtering. The recommendations of the SENIAM project indicate a time window of 0.25 to 0.5 seconds [21]. It corresponds to the choice of cut-off frequency of the low-pass filter of 2 to 4 Hz. This critical parameter choice depends on the task dynamics. Indeed, this choice of cut off frequency is a trade off between how fast the fluctuations in amplitude can be and how reliable the estimate of the amplitude is. A low cut off frequency gives a more reliable estimate of the amplitude, but cannot capture fast changes, while higher cut off frequency results in noisier output but captures fast contractions. The cut off frequency mentioned in the SENIAM recommendations is 2 Hz for slow motions, and 6 Hz for fast motions [21]. In Hill-type model, this cut off frequency should be carefully set depending on the type of task because the envelope of the estimated force is almost determined by this low-pass filtering step. The normalized EMG is processed with the following activation dynamics, which mainly captures the delay from muscle activation to mechanical output.

#### Activation dynamics

**Figure 1:**
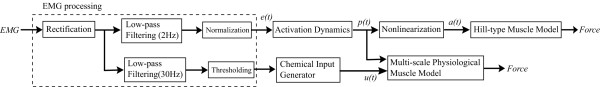
A flowchart of EMG-force estimation by the Hill-type model and multiscale physiological muscle model.

The process of transforming the normalized, rectified and filtered EMG, referred as *e*(*t*) to neural activation *p*(*t*), is called the activation dynamics as shown in the Figure 1 flowchart. When a muscle fiber is activated by a single action potential (AP), the muscle generates a twitch response. This delayed response can be approximated by a critically damped linear second-order differential system [8]. Its recursive discrete form is as follows in Eq. 1 to calculate *p*(*t*_*k*_).

(1) $$p(t_{k})=\mathrm{\gamma e}(t_{k}-d)-\beta_{1}p(t_{k-1})-\beta_{2}p(t_{k-2})$$

where d is the electromechanical delay and *γ*, *β*_1_ and *β*_2_ are the coefficients that define the second-order dynamics. To obtain a positive stable solution, a set of constraints are employed, i.e. *β*_1_=*C*_1_+*C*_2_,*β*_2_=*C*_1_*C*_2_ where |*C*_1_|<1,|*C*_2_|<1. In addition, the gain of this filter should be maintained by ensuring *γ*−*β*_1_−*β*_2_=1 as described in [8].

#### Nonlinearization of muscle activation for nonlinear Hill model

Many researchers assume that the above *p*(*t*) is a reasonable approximation of muscle activation. However, a nonlinear relationship has been reported between individual muscle EMG and the joint moment for some muscles, especially at lower forces [14]. In studies on single motor units, multiple APs cause succesive twitch responses. If the time between APs decreases, twitches start to merge and the muscle force increases steadily. However, at high frequency we reach maximal contraction, where force development saturates even if the frequency increases. This induces a nonlinear relationship between the frequency and force for single motor units [8].

This modification was thus recently introduced to correspond to the mismatch of Hill model estimation at low activation levels. Indeed, in the classical linear Hill model, neural activation *p*(*t*) is directly treated as muscle activation *a*(*t*) without the nonlinear conversion. When we refer to the modified nonlinear Hill model, the following conversion from neural activation *p*(*t*) to muscle activation *a*(*t*) is performed as in Figure 1. As a simple and adequate solution, Lloyd and Besier [9] proposed the following formulation:

(2) $$a(t)=\frac{e^{\text{Ap}(t)}-1}{e^{A}-1}$$

where A is a constant parameter for the nonlinear shape factor allowed to vary between -3 and 0, with *A*=−3 being highly exponential and *A*=0 being linear.

#### Hill-type contraction dynamics

**Figure 2:**
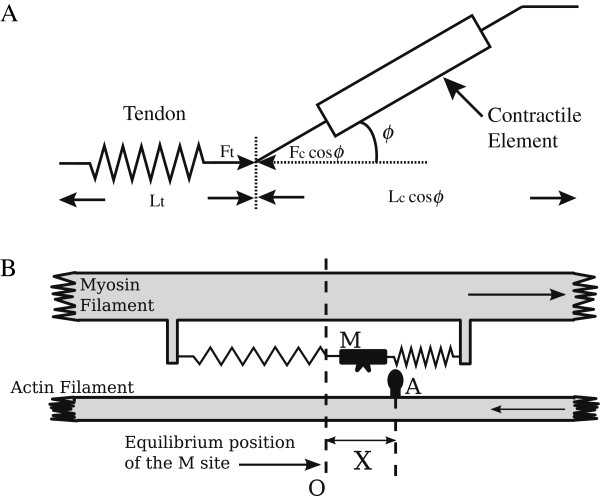
**Muscle models. (A)** Muscle-tendon macroscopic model. **(B)** Huxley sliding filaments model.

The muscle-tendon unit is modeled as a contractile element in series with an elastic tendon, as shown in Figure 2(A) as same as the general Hill model [12]. The tendon element in series was modeled as having a linear stiffness *k*_*t*_ of 180 N/mm [22] as the parameter in human triceps surae muscle-tendon complex. *ϕ* is the pennation angle between the tendon and muscle fibers. Moreover, in this paper, we assess the EMG-force relationship in isometric conditions. Then, only concentric contraction is considered where a contractile element is always shortening during contraction. The parallel elastic element is not included, since its length does not change during isometric contraction, which is assumed in this study. In addition, we would include the parallel element effect in joint level modeling rather than the muscle level when we apply the proposed approach in non-isometric condition. This common macroscopic structure as shown in Figure 2(A) is used for the three types of modeling, only the contractile element dynamics change.

The Hill-type muscle model is used to estimate the force *F*_*c*_(*t*) generated by the contractile element with the general form:

(3) $$F_{c}(t)=a(t)f_{l}(\varepsilon_{c})f_{v}({\dot{\varepsilon }}_{c})F^{m}$$

where *ε*_*c*_ is the strain of the contractile element, *f*_*l*_(*ε*_*c*_) and $f_{v}({\dot{\varepsilon }}_{c})$ are the normalized force-length and force-velocity relationships, respectively. *F*^*m*^ is the maximum isometric muscle force.

The force-length relationship shows a Gaussian curve around the optimal length for which the muscle generates the maximum force [12]:

(4) $$f_{l}(\varepsilon_{c})=\exp\left\{-{\left(\frac{\varepsilon_{c}}{b}\right)}^{2}\right\}$$

where *b* is a constant parameter. $f_{v}({\dot{\varepsilon }}_{c})$ represents the relationship between the velocity and the normalized force. The muscle contracts at its maximum velocity *v*_*max*_ without load and slows down as the load increases. In the case of concentric contraction, this relationship can be formulated as follows:

(5) $$f_{v}({\dot{\varepsilon }}_{c})=\frac{V_{\text{sh}}(v_{\text{max}}+L_{c0}{\dot{\varepsilon }}_{c})}{V_{\text{sh}}v_{\text{max}}-L_{c0}{\dot{\varepsilon }}_{c}}$$

where *L*_*c*0_ is the length of contractile element at rest position and *V*_*sh*_ is a constant parameter. The *b* and *V*_*sh*_ parameters were obtained from [23,24]. At each time step, the fiber velocity should be solved and the muscle fiber length is computed by forward integration using Runge-Kutta algorithm [8]. Since the value of *ε*_*c*_ changed, the calculation should continue iteratively until the end of the input time series of *a*(*t*).

The muscle tendon parameters were adopted from Delp [25]. For gastrocnemius, the parameters are averages for two heads (med/lat). Only EMG of the medial head is measured, but the force of both heads is estimated using the sum of maximum force of two heads through *F*^*m*^. *F*^*m*^ and pennation angle *ϕ* are also obtained from [25]. However, in the following, the final result is normalized by the maximum contraction, so the effect of these parameters is not relevant. Parameters of muscle model are detailed in Table 1.

**Table 1:** Muscle model parameters

	**Parameter**	**Unit**	**Value**
	*b*	-	0.5
	*V*_*sh*_	-	0.3
	*L*_*c*0_(*GAS*)	*cm*	5.1
Hill	*L*_*t*0_(*GAS*)	*cm*	40
	Pennation *ϕ*(GAS)	Degrees	14
Common	*F*_*m*_(GAS)	*N*	1600
parameters	*L*_*c*0_(*SOL*)	*cm*	3.0
	*L*_*t*0_(*SOL*)	*cm*	26.8
	Pennation *ϕ*(SOL)	Degrees	30
	*F*_*m*_ (SOL)	*N*	2830
	*k*_*t*_	*N*/*mm*	180
	*A* (subject1)	-	-1.7
Nonlinear	*A* (subject2)	-	-1.2
Hill	*A* (subject3)	-	-1.7
	*A* (subject4)	-	-1.5
	*U*_*c*_	1/s	5
Physiological	*U*_*r*_	1/s	10
model	*k*_*m*_	*N*/*m*	*F*_*m*_×20

Only A parameter for nonlinear Hill model is identified for each subject.

### Physiological muscle model

We breifly describe the main points of the multi-scale physiological muscle model. It integrates the characteristics both of macroscopic and microscopic muscle dynamics. The details of this modeling are given in [19]. This model was originally designed to represent the muscle response under FES and is applied to voluntary muscular force estimation based on EMG in this paper.

#### Generation of chemical input

**Figure 3:**
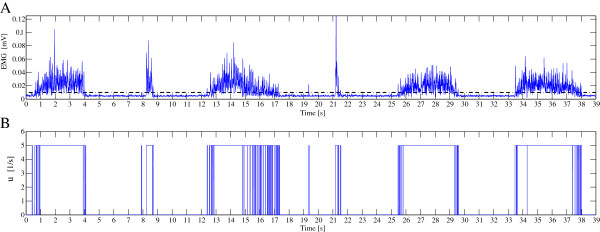
**Generated chemical input. (A)** Filtered rectified EMG signal. **(B)** Chemical input by thresholding for SOL.

The physiological model is composed of two inputs as shown in Figure 1. One is the recruitment rate *α* which determines the percentage of recruited muscle fibers. The recruitment rate *α* has the same physiological meaning as the neural activation *p*(*t*) in Hill-type model. Then normalized *p*(*t*) without the nonlinearization was directly used for *α*. The second input for physiological muscle model is the chemical signal *u*. It represents the underlying physiological processes between the contraction and relaxation states independent from recruitment. Muscle contraction is initiated by an AP along the muscle fiber membrane, which goes deeply into the cell through T-tubules. It causes calcium release which induces the contraction process when the concentration goes above a threshold and is sustained until the concentration decreases below this threshold again [26]. Hatze [27] gives an example of calcium dynamics [ *Ca*^2+^] modeling. The contraction-relaxation cycle is then triggered by the [ *Ca*^2+^] to be defined, and associated with two phases: i) contraction and ii) relaxation. We use a delayed model to take into account the propagation time of the AP and an average delay due to the calcium dynamics. It corresponds to the electromechanical delay for *e*(*t*) into *p*(*t*) conversion in Hill-type approach. For chemical input generation, the rectified EMG was low-pass filtered with a 30 Hz cut-off frequency. Then, the chemical input *u*(*t*) was created by thresholding the extracted EMG signals as shown in Figure 3. The signal measured in EMG is the summation of the APs of all different MUs. However, as we are trying to deal with one muscle model for the whole muscle force estimation, the EMG signal was considered as a representative average triggering signal. The thresholding can be assumed to reflect a muscle cell’s all-or-nothing response corresponding to the above chemical nature of the muscle contraction process.

Thus, the chemical threshold consists basically in extracting the contraction time duration of the muscle and to indicating if the muscle is in contraction or not. The level of activation itself is managed by recruitment rate which is classically used in EMG usage for Hill model. Thus, especially for the low activation state, even if the chemical input is inaccurately determined, its effect is low since the recruitment activation p(t) is very low during this period. And for the higher activation state, the contraction period is accurately captured by this method. As long as the threshold is taken little above the baseline, the threshold is less sensitive as long as it gives the picture of contraction/relaxation state. The parameters for chemical input generation was obtained from [18,28].

#### Sarcomere scale

All the sarcomeres are assumed to be identical, and the deformation of both sarcomere and muscle is proportional. If *S* is the sarcomere length, we can write (*S*−*S*_0_)/*S*_0_=(*L*_*c*_−*L*_*c*0_)/*L*_*c*0_=*ε*_*c*_.

Huxley proposed that a cross-bridge between actin filaments and myosin heads could exist in two biochemical states, i.e. attached and detached states as in Figure 2(B). Filaments sliding is the result of interactions between the myosin cross-bridges and the thin actin filaments. The cross-bridges reversibly bind to actin and produce a mechanical impulse, which in turn results in force transmission along the filaments. M and A in the figure represents the myosin head and actin binding site respectively.

One myosin head can only attach to one actin site. Then the dynamics of the fraction *n*(*y*,*t*) of the attached cross bridges is given by

(6) $$\frac{\mathrm{\partial n}}{\mathrm{\partial t}}+\frac{S_{0}}{h}{\dot{\varepsilon }}_{c}\frac{\mathrm{\partial n}}{\mathrm{\partial y}}=f(y,t)\left[\;1-n(y,t)\right]-g(y,t)n(y,t)$$

where *h* is the maximum elongation of the myosin spring, *x* is the distance and *y* the normalized distance between the actin binding site and the myosin head: $y=\frac{x}{h}$. *n*(*y*,*t*) is a distribution function representing the fraction of attached cross bridges relative to the normalized relative position *y*. To ensure a one way displacement, this attachment is considered possible only when *y* is between 0 and 1. $S_{0}{\dot{\varepsilon }}_{C}$ represents the velocity of the actin filament relative to the myosin filament. *f* and *g* denote the rate functions of attachment and detachment, respectively.

Several *f* and *g* functions have been defined and recently a chemical input was introduced by Bestel-Sorine [18] to modify the ability of the cross bridge to attach or not. They also proposed that these rates depend on the relative velocity between actin and myosin. Indeed, the higher the velocity is, the greater the probability to break bridges is. *f* and *g* can thus be defined by: *During the contraction phase*

(7) $$\left\{\;\begin{array}{l} f(y,t)=U_{c}\\ g(y,t)=U_{c}+\left|{\dot{\varepsilon }}_{c}\right|-f(y,t) \end{array}\right)$$

During the relaxation phase

(8) $$\left\{\;\begin{array}{l} f(y,t)=0\\ g(y,t)=U_{r}+\left|{\dot{\varepsilon }}_{c}\right| \end{array}\right)$$

*U*_*c*_ and *U*_*r*_ are the chemical kinetics levels under contraction and relaxation phases respectively. That can be resumed as below

(9) $$\begin{array}{l} u(t)=\Pi_{c}(t)U_{c}+(1-\Pi_{c}(t))U_{r}\\ \Pi_{c}(t)=1\;\text{during contraction, 0 else}\\ (f+g)(y,t)=u(t)+\left|{\dot{\varepsilon }}_{c}\right| \end{array}$$

To complete the description at the sarcomere scale, we assume that the force generated by one attached cross bridge is modeled by a linear spring with constant stiffness. All cross bridges are parallel so the global stiffness and force generated by the whole sarcomere is proportional to the number of formed bridges. Let *k*_0_ (Nm ^−1^) denote the maximum stiffness obtained when all the available bridges are attached. Let *ξ*(*y*,*t*) denote the elongation of a cross bridge due to the contribution of the global extension of the sarcomere, where *ε*_*c*_(0) is the initial value, and to the local distribution of elongations *y*:

(10) $$\xi (y,t)=y+\frac{S_{0}}{h}\left(\varepsilon_{c}(t)-\varepsilon_{c}(0)\right)$$

The stiffness and force generated by a muscle sarcomere is obtained by computing the first and second moment of the distribution *n*(*ξ*(*y*,*t*),*t*) [17]. The two first moments are defined as:

(11) $$\begin{array}{l} \;\;\;k_{s}(t)=k_{0}\overset{+\infty }{\underset{-\infty }{\int }}n(\xi (y,t),t)\text{dy}\\ F_{s}(t)=k_{0}h\overset{+\infty }{\underset{-\infty }{\int }}\xi (y,t)n(\xi (y,t),t)\text{dy} \end{array}$$

From Eq. 6, it can be rewritten as follows:

(12) $$\begin{array}{l} {\dot{k}}_{s}=-\left(f+g\right)(y,t)k_{s}+k_{0}f(y,t)\\ {\dot{F}}_{s}=-\left(f+g\right)(y,t)F_{s}+k_{s}S_{0}{\dot{\varepsilon }}_{c}+\frac{1}{2}k_{0}\text{hf}(y,t) \end{array}$$

Contrary to Zahalak approximation, we do not make any assumption on the distribution, rather the chosen f and g functions allows for a straightforward computation of the stiffness and the force generated by the contractile element. It provides a computational effective model that however relies on Huxley theory.

#### Myofiber and muscle scale

This set of differential equations can be easily extended to the whole muscle fiber considering that each fiber is composed of identical sarcormeres in series. Then $k_{f}=k_{s}\frac{S_{0}}{L_{c0}}$ and *F*_*f*_=*F*_*s*_. In addition, the maximum available cross bridges varied depending on the relative length of the contractile element. This is known as the force-length relationship (*f*_*l*_(*ε*_*c*_)). Contrary to previous studies where this effect is introduced at the macroscopic level, we take into account this relation at the microscopic scale [17]. Indeed, this relation is directly linked to the maximum available actin and myosin sites [16]. For the stiffness *k*_*f*_ and the force *F*_*f*_ at the fiber scale including Eq. 9, we get:

(13) $$\begin{array}{l} {\dot{k}}_{f}=-\left(u+\left|{\dot{\varepsilon }}_{c}\right|\right)k_{f}+\frac{S_{0}}{L_{c0}}k_{0}\Pi_{c}(t)U_{c}f_{l}(\varepsilon_{c})\\ {\dot{F}}_{f}=-\left(u+\left|{\dot{\varepsilon }}_{c}\right|\right)F_{f}+k_{f}L_{c0}{\dot{\varepsilon }}_{c}+\frac{1}{2}k_{0}h\Pi_{c}(t)U_{c}f_{l}(\varepsilon_{c}) \end{array}$$

Next, we introduced the recruitment process at muscle scale. At each contraction phase, the recruitment ratio is updated but remains constant during its phase itself. Let *k*_*c*_ and *F*_*c*_ denote the stiffness and the force for whole contractile element, and *N* the number of all MUs. The recruited number is written as *αN* using recruitment ratio *α*. Finally the complete model of contractile element can be written as a set of differential equations:

(14) $$\begin{array}{l} {\dot{k}}_{c}=-(u+\left|{\dot{\varepsilon }}_{c}\right|)k_{c}+\alpha k_{m}\Pi_{c}(t)U_{c}\\ {\dot{F}}_{c}=-(u+\left|{\dot{\varepsilon }}_{c}\right|)F_{c}+\alpha F_{m}\Pi_{c}(t)U_{c}+k_{c}L_{c0}{\dot{\varepsilon }}_{c} \end{array}$$

where *k*_*m*_=*S*_0_*Nk*_0_*f*_*l*_(*ε*_*c*_)/*L*_*c*0_,*F*_*m*_=*Nk*_0_*hf*_*l*_(*ε*_*c*_)/2. *k*_0_ (Nm ^−1^) is the maximum stiffness obtained when all the available bridges are attached.

Recruitment rate is thus mixed with the dynamics of the contraction relaxation cycle providing a wider range of mechanical responses than Hill approaches. Compared to Zahalak approximation, we provide a two input model with intricate dynamics due to both internal muscle dynamics and recruitment rate dynamics in a straightforward way.

#### Computation of the whole dynamics

For the macroscopic representation, the same configuration with Hill models as in Figure 2(A) is used including the muscle tendon parameters. The contractile element is replaced with the above nonlinear differential equations.

The dynamics of the contractile element coupled with the tendon in series should also comply with the following equation:

(15) $${\dot{F}}_{c}=k_{t}L_{t0}{\dot{\varepsilon }}_{t}/\mathrm{cos\phi}=k_{t}/\mathrm{cos\phi}(L_{0}\dot{\varepsilon }-L_{c0}{\dot{\varepsilon }}_{c})$$

In order to have one solution for two equations of ${\dot{F}}_{c}$, we must verify that: *k*_*t*_*L*_*c*0_/*cosϕ*+*k*_*c*_*L*_*c*0_−*F*_*c*_>0. The differential of elongation *ε*_*c*_ is computed with the following equation:

(16) $${\dot{\varepsilon }}_{c}=\frac{k_{t}L_{0}\dot{\varepsilon }/\mathrm{cos\phi}+F_{c}u-\alpha F_{m}\Pi_{c}(t)U_{c}}{k_{t}L_{c0}/\mathrm{cos\phi}+k_{c}L_{c0}-S_{{\dot{\varepsilon }}_{c}}F_{c}}$$

$S_{{\dot{\varepsilon }}_{c}}$ is the sign of ${\dot{\varepsilon }}_{c}$. From the condition: *k*_*t*_*L*_*c*0_/*cosϕ*+*k*_*c*_*L*_*c*0_−*F*_*c*_>0, $S_{{\dot{\varepsilon }}_{c}}$ can be obtained from the sign of these terms: $k_{t}L_{0}\dot{\varepsilon }/\mathrm{cos\phi}+F_{c}u-\alpha F_{m}\Pi_{c}(t)U_{c}$. Then we can compute ${\dot{k}}_{c}$ and ${\dot{F}}_{c}$ with (14). The internal state vector of this system should be set as $x=\left[\;\begin{array}{lll} k_{c} & F_{c} & \varepsilon_{c} \end{array}\right]$.

### Experimental measurement and data processing

Four healthy volunteers (2 males and 2 females, age =30.8±1.3 years, mass =66.5±16.0 kg, and stature =1.7±0.13 m) participated in the study after signing an informed consent form. The study was approved by the Agence française de sécurité sanitaire des produits de santé (Afssaps) committee for persons’ protection managed by CHRU Montpellier (2011-A01033-38), dedicated to the study of the lower limb biomechanics within ANR SoHuSim project.

**Figure 4:**
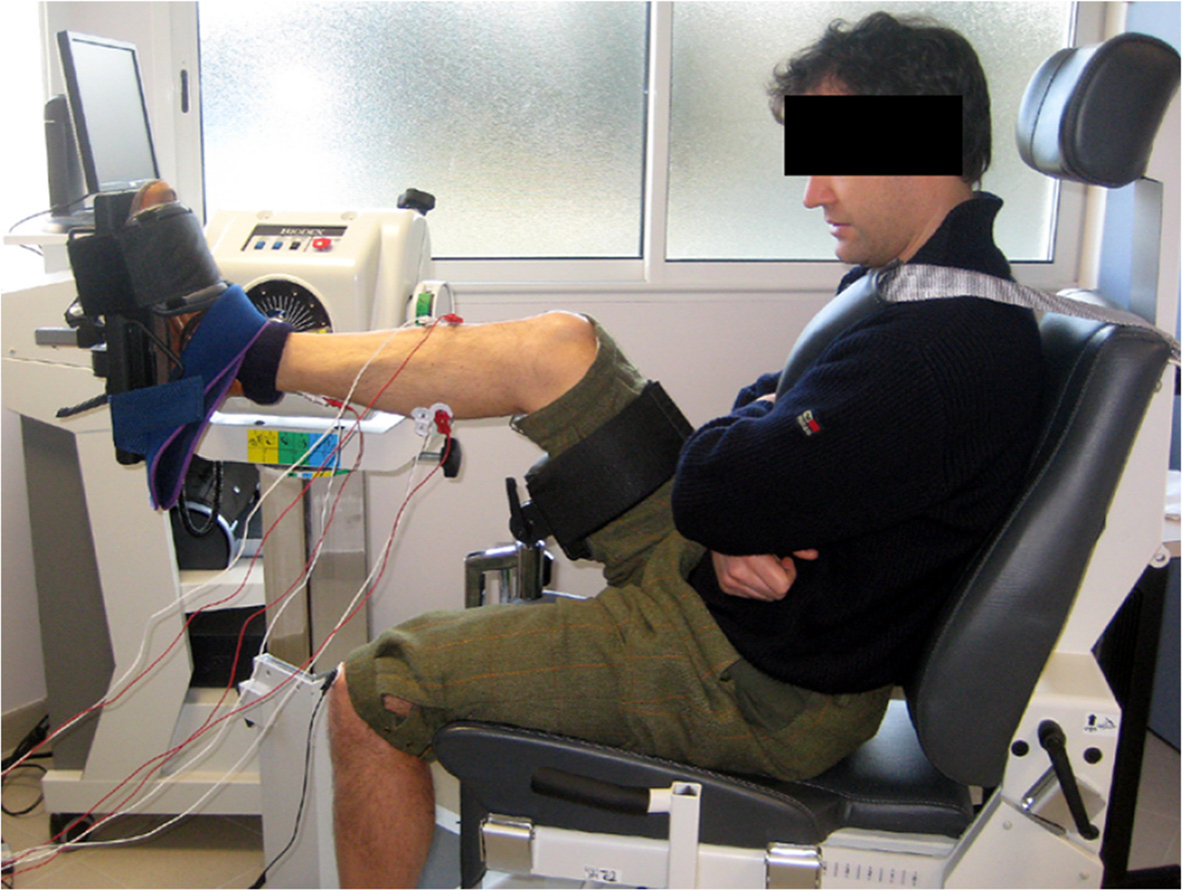
**Appearance of the experiment.** The torque around the ankle joint was measured along with EMG of medial gastrocnemius (GAS) and soleus (SOL) during voluntary plantar flexion.

The subjects were seated on a chair with their right foot attached to a Biodex dynamometer (Biodex Medical Systems Inc., New York, USA) as shown in Figure 4. The setting was 90° for the ankle joint and 110° for the knee joint, while straps were used on the pelvis and shoulders to secure the subjects’ position on the chair. The axis of the dynamometer was aligned with the ankle’s rotation axis. The torque around the ankle joint was measured when it was voluntarily generated for the plantar flexion. For EMG measurements, bipolar surface Ag/AgCl-electrodes were placed on the muscle belly of the medial gastrocnemius (GAS) and soleus (SOL) with an inter-electrode distance of 20 *mm*. The reference electrode was placed on the left patella. Synchronous acquisition of the force and differential EMG signal was performed with a sampling frequency of 2048 *Hz* by the EMG100C amplifier and Biopac MP100 system (Biopac Systems, Inc., Santa Barbara, USA).

In this preliminary test, we focused on the isometric condition and tried to compare the output force on a normalized scale against the output during MVC. The common parameters between three approaches were set at the same value as summarized in Table 1. In order to maintain similar conditions, the common macroscopic model as in Figure 2(A) was used for all the three approaches. Only the dynamics representation of the contractile element was replaced. *A* was the only parameter identified for each subject. *A* was obtained by minimizing the root mean square difference between the measured and the model estimation at MVC. Other muscle parameters except *A* are taken from literatures as explained in the model section.

The estimated muscle force is multiplied by the moment arm in order to obtain the torque. The moment arm was estimated from the Hawkins data [29] from the joint angle in the measured condition. The moment arms for MG and SOL are 0.0515 [m] and 0.0464 [m], respectively. The contribution ratio was calculated using the values reported by Delp [25]. This was obtained as the maximum force by the moment arm considering the pennation angle. The resulting ratio is MG 0.41 vs SOL 0.59. The SUM in the estimation result is plotted using the ratio as the sum of the two muscles.

The generated input command *u*(*t*) and activation rate *α* were given to the contractile element of the physiological model and the active stiffness *k*_*c*_ and the muscle force *F*_*c*_ were computed using Eq. 14. The same value for *F*_*m*_ for the three approaches were used. *k*_*m*_ is proportional to *F*_*m*_ from the condition in Eq. 14. It was multipied by 20 times, as shown in Table 1, based on the knowledge that the muscle active stiffness reaches the tendon stiffness at approximately maximum contraction [30]. However, this muscle stiffness parameter does not influence much the force output in isometric condition.

## Results

The predicted joint torque based on EMG signals was compared with the directly measured torque of the ankle joint for the plantar flexion. The comparison was made against the case in MVC in normalized scale. Thus, we avoided the situation effected by the subject-specific difference regarding muscle strength, which is significant for torque estimation in absolute scale.

**Figure 5:**
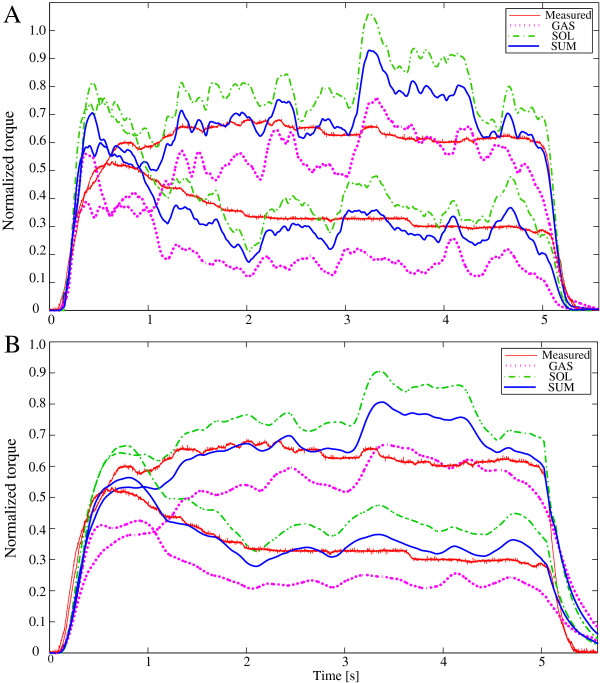
**Normalized estimated torques for 30% and 70% of MVC.** (red: measured, magenta: estimated by the model of GAS, green: estimated by the model of SOL, blue: sum of both models) **(A)** result obtained by the modified Hill model. **(B)** result obtained by the full physiology based model.

First, the normalized torques for 30% and 70% of MVC estimated by the nonlinear Hill model and multi-scale model were computed as in Figure 5. The normalized data of measured torque (red), normalized estimated torque of SOL (green), normalized estimated torque of GAS (magenta) and the sum of two muscles (blue) are plotted. The obtained RMS errors for the first two subjects are given in Table 2 for both contraction levels and by both estimation approaches. The estimation results of physiology based model show the smooth transition compared to the estimation by Hill model keeping the realistic torque development as in the measured torque. In addition, especially for low activation levels, RMS errors in the physiology based model showed better accuracy. Note that the modified Hill model has already the nonlinearization conversion to correspond to a low activation level. The proposed physiological model doesn’t include such empirical modifications.

**Table 2:** RMS errors between the measured and estimated results in 30% and 70% of MVC

	**Nonlinear Hill**		**Full physiology**	
**Subject**	**30%**	**70%**	**30%**	**70%**
1	0.0852	0.089	0.0365	0.0848
2	0.047	0.106	0.0307	0.0871

RMS errors of 0.1 correspond to 10% error.

**Figure 6:**
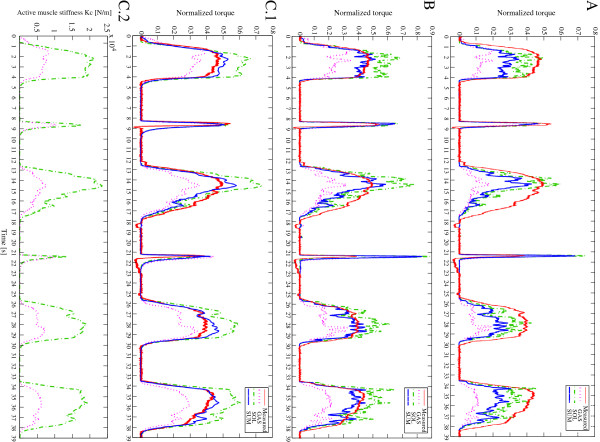
**Normalized estimated torques for random contraction.** (red: measured, magenta: estimated by the model of GAS, green: estimated by the model of SOL, blue: sum of both models) **(A)** result obtained with the linear Hill model. **(B)** result obtained with the modified nonlinear Hill model. **(C.1)** result obtained with the multi-scale physiology based model. **(C.2)** The corresponding active muscle stiffness *k*_*c*_ estimated with the proposed model.

Next, in order to confirm the estimation ability for both fast-short and slow-long contraction, random contractions including these two types of contraction were requested to all subjects. The generated chemical inputs are shown in Figure 3. Normalized torques estimated by linear Hill model (A), nonlinear Hill model (B) and multi-scale physiology based model (C.1) are depicted in Figure 6. In the physiological model, active stiffness and strain of the contractile element are explicitly defined in differential equations, then it can be estimated along with the muscle force. The active muscle stiffness *k*_*c*_ is shown at Figure 6(C.2). The results of linear Hill model is computed to show the contribution of the nonlinear conversion. As we observe the difference between them in Figure 6(A,B), nonlinear Hill model estimation is improved especially for low level contractions. However, the errors for fast-short contraction remained high. In contrast, the estimation result as in Figure 6(C.1) with the proposed method demonstrates the nice estimation for all conditions of contractions.

The obtained RMS errors for the four subjects are given in Table 3 for both contraction types and by three estimation approaches. In addition, mean error of the peak torque for fast-short contraction are given. Average results is also reported as *mean*±*standard* deviation over all the subjects. Average error was 16.9*%* with the linear Hill model, 9.3*%* with the nonlinear Hill model. In contrast, the error in the multi-scale model was 6.1*%* while maintaining the uniform estimation performance in both fast and slow contraction speeds. Mean peak error for fast-short contraction are 21.9*%* with the linear Hill model, 17.6*%* with the nonlinear Hill model. In contrast, the error in the multi-scale model was 5.8*%*. In general, results estimated by physiology based model show the best performance both in fast-short and slow-long contractions in these random tests, as reported in Table 3.

**Table 3:** RMS errors between the measured and estimated results in random contraction

**Method**	**Error type**	**Subject1**	**Subject2**	**Subject3**	**Subject4**	***Mean±SD***
	RMS (whole)	0.117	0.153	0.195	0.212	0.169±0.043
Linear	RMS (slow-long)	0.133	0.186	0.233	0.252	0.201±0.053
Hill	RMS (fast-short)	0.0508	0.0435	0.0706	0.0909	0.064±0.021
	Peak error (fast)	0.188	0.050	0.30	0.338	0.219±0.129
	RMS (whole)	0.063	0.0681	0.126	0.116	0.093±0.032
Nonlinear	RMS (slow-long)	0.0585	0.0795	0.0986	0.136	0.093±0.033
Hill	RMS (fast-short)	0.0806	0.0318	0.167	0.0682	0.087±0.057
	Peak error (fast)	0.274	0.0583	0.317	0.057	0.176±0.138
	RMS (whole)	0.0499	0.0373	0.0531	0.102	0.061±0.028
Physiological	RMS (slow-long)	0.0427	0.0418	0.0558	0.103	0.061±0.029
model	RMS (fast-short)	0.0742	0.0242	0.0472	0.101	0.062±0.033
	Peak error (fast)	0.0354	0.013	0.0536	0.131	0.058±0.051

## Discussion

Hill-type muscle is a phenomenological model based on experimental facts with no link to the microscopic physiology. First, even if the estimation performances are equivalent, it is meaningful to understand and capture the muscle dynamics with a more detailed representation. Indeed, the same kind of force estimation could be obtained with the newly proposed physiological model using voluntary EMG signals.

Second, previous work by Perreault [13] reported that Hill modeling errors were large for different firing frequencies and greatest at low MU firing rates. The classical linear Hill model was used. They suggested that more physiological coupling between activation and force-velocity properties may help to deal with these issues [13]. Thus, the aim of introducing new approach based on a physiologically detailed model [19] was to develop EMG-force estimation with natural velocity dependency incorporating microscopic cross-bridge dynamics on top of recruitment dynamics.

In general, the estimation accuracy of the proposed method is better than that of the linear and nonlinear Hill-type approach as in the obtained result regarding RMS errors. In addition, uniform accuracy was obtained for different types of contractions in the proposed method. In particular, the proposed model is able to properly deal with both slow and fast contraction speeds. When considering the Hill model results, Figure 6(A,B) shows largest errors for the estimation of fast-short contractions. The reason of this error could be explained as follows: the signal measured in EMG is the summation of the APs of all different MUs. Even in the fast-short contraction, the amplitude of EMG itself does not reflect differences except duration with the same level as for slow-long contraction. However, the resulting fast-short contraction force is actually much less than that of slow-long contraction. This means that there is sometimes hysteresis regarding the neural command. In Hill approach, muscle activation *a*(*t*) is proportional to the resulting force. It therefore does not include the effect of the time hysteresis in contraction [13], thus Hill model can not accurately represent both short and long-term contractions simultaneously with only one choice of cut-off frequency in EMG low-pass filtering. In the proposed approach, the derivative of the contraction force is directly given by the neural command and it brings time hysteresis in force generation. It is interesting to note that this kind of uniform accuracy for different types of contractions was appeared along with the introduction of cross-bridge dynamics.

In addition, it is known that there is a nonlinear relationship between frequency during contraction and force for single MUs [14]. In modified Hill model, this frequency dependency is offset only by the nonlinear conversion. This nonlinearization was recently proposed to modify the classical Hill model. This process was originally not introduced in the so-called Hill model. In fact, this modification brings much better estimation, especially at lower forces. However, even with the modification, it is not a time function so it still can not correspond to the different muscle contraction speeds. In Hill-type approach, neural activation *p*(*t*) is dominantly imposed by low-pass filtering with a 2–10 Hz cut-off frequency. The choice of cut-off frequency is very sensitive to the dynamics of *p*(*t*). *p*(*t*) is more or less proportional to the resulting force. Therefore, it is advised that the choice of cut off frequency should be carefully selected depending on the type of task in Hill-type modeling. In this study, a 2 Hz cut-off frequency was used according to the SENIAM recommendation. Sometimes by changing the cut-off frequency for different types of contraction, better results can be obtained with Hill-type model. However, in any case, the expected motion by subject is unknown in advance for practical use of EMG-force estimation, so it is difficult to have an appropriate cut-off frequency in advance. Moreover it assumes that the subject stays in the same kind of motion dynamics, that is not always the case. In the proposed model, the nonlinear activation property coming from force-velocity dependency is internally integrated in the model dynamics.

Furthermore, the physiological model satisfies the well established properties observed for the muscle’s behavior: i) the force-length relation is included in the definition of Eq. 13, ii) the force-velocity relationship can be expressed in isotonic and tetanic conditions. Before isotonic contraction could occur, the muscle contracts in isometric conditions until the force generated by the muscle balances the imposed one. Isotonic contraction then becomes possible. Assuming ${\dot{F}}_{c}=0$ in Eq. 14, the following equation may be formulated: $F_{c}=(\alpha F_{m}U_{c}+k_{c}L_{c0}{\dot{\varepsilon }}_{c})/(U_{c}+\left|{\dot{\varepsilon }}_{c}\right|)$.

At the beginning of this phase (t = 0), *k*_*c*_(0)≠0 but it depends on the initial isometric contraction phase. If we define $A(0)=\frac{L_{c0}k_{c}(0)}{U_{c}\alpha F_{m}},B=\frac{1}{U_{c}}$, in concentric contraction where ${\dot{\varepsilon }}_{c}<0$, we get a Hill type force-velocity relation:

(17) $$F_{c}=\alpha F_{m}\frac{1+A(0){\dot{\varepsilon }}_{c}}{1-B{\dot{\varepsilon }}_{c}}$$

This correspondence can be found in Eq. 5. It can be verified that our physiological muscle model integrates the force-velocity relation naturally when considering the actin-myosin cross bridge. Finally, this improvement in estimation accuracy is in line with the suggestion put forward in a previous work [13] on introducing crossbridge model.

Future study will be focused on simulations with multiple sets of the proposed model corresponding to multiple MUs in one muscle considering size principal, fast/slow fibers. However, for the application of voluntary EMG-force estimation, we should keep the simplicity as Hill-type model. Future work will also focus on increasing the number of experimental tests and we should develop an algorithm to statistically determine if a muscle is turned on/off regarding the chemical thresholding. In addition, the further interpretation of neuromuscular system both in voluntary and FES activations should be pursued. We have already performed the study on the identification and validation of the physiological model in in-vivo rabbit experiment [31] and in paraplegic subjects including non-isometric situation under FES [32]. In order to be applied to a broader range of clinical situations, further investigations in isokinetic and isotonic cases should be carried out.

## Conclusion

In this paper we presented a method that allows estimation of muscle force from EMG signals with a multi-scale physiology based model with a link to underlying microscopic filament dynamics. The experimental results highlight the feasibility of the torque estimation and its comparison with Hill-type models using the same EMG signals. This is the first report on EMG-force estimation based on a multi-scale physiology model integrating Hill-type and microscopic cross bridge representations. The proposed method features: 

•a novel physiologically detailed model for EMG-force estimation in the place of a phenomenological Hill-type muscle model,

•the estimation improvement especially for different types of contraction incorporating dual dynamics of recruitment and microscopic cross-bridge formation toward coupled activation-velocity relationship.

## Competing interests

The authors declare that they have no competing interests.

## Authors’ contributions

MH proposed and built the structure of EMG-force estimation with a multi-scale model and wrote the first draft. DG was in charge of the physiological muscle modeling and proof reading. MH performed the scientific computation and experiments. All authors read and approved the final manuscript.
